# Differential Requirements for Mediator Complex Subunits in *Drosophila melanogaster* Host Defense Against Fungal and Bacterial Pathogens

**DOI:** 10.3389/fimmu.2020.478958

**Published:** 2021-03-05

**Authors:** Chuqin Huang, Rui Xu, Samuel Liégeois, Di Chen, Zi Li, Dominique Ferrandon

**Affiliations:** ^1^Sino-French Hoffman Institute, Guangzhou Medical University, Guangzhou, China; ^2^Université de Strasbourg, UPR 9022 du CNRS, Strasbourg, France

**Keywords:** mediator complex, *Drosophila* innate immunity, Toll pathway, host defense against fungi and bacteria, RNA interference, survival to infection, humoral immune response

## Abstract

The humoral immune response to bacterial or fungal infections in *Drosophila* relies largely on a transcriptional response mediated by the Toll and Immune deficiency NF-κB pathways. Antimicrobial peptides are potent effectors of these pathways and allow the organism to attack invading pathogens. Dorsal-related Immune Factor (DIF), a transcription factor regulated by the Toll pathway, is required in the host defense against fungal and some Gram-positive bacterial infections. The Mediator complex is involved in the initiation of transcription of most RNA polymerase B (PolB)-dependent genes by forming a functional bridge between transcription factors bound to enhancer regions and the gene promoter region and then recruiting the PolB pre-initiation complex. Mediator is formed by several modules that each comprises several subunits. The Med17 subunit of the head module of Mediator has been shown to be required for the expression of *Drosomycin*, which encodes a potent antifungal peptide, by binding to DIF. Thus, Mediator is expected to mediate the host defense against pathogens controlled by the Toll pathway-dependent innate immune response. Here, we first focus on the Med31 subunit of the middle module of Mediator and find that it is required in host defense against *Aspergillus fumigatus*, *Enterococcus faecalis*, and injected but not topically-applied *Metarhizium robertsii*. Thus, host defense against *M. robertsii* requires *Dif* but not necessarily *Med31* in the two distinct infection models. The induction of some Toll-pathway-dependent genes is decreased after a challenge of *Med31* RNAi-silenced flies with either *A. fumigatus* or *E. faecalis*, while these flies exhibit normal phagocytosis and melanization. We have further tested most Mediator subunits using RNAi by monitoring their survival after challenges to several other microbial infections known to be fought off through DIF. We report that the host defense against specific pathogens involves a distinct set of Mediator subunits with only one subunit for *C. glabrata* or *Erwinia carotovora carotovora*, at least one for *M. robertsii* or a somewhat extended repertoire for *A. fumigatus* (at least eight subunits) and *E. faecalis* (eight subunits), with two subunits, Med6 and Med11 being required only against *A. fumigatus*. *Med31* but not *Med17* is required in fighting off injected *M. robertsii* conidia. Thus, the involvement of Mediator in *Drosophila* innate immunity is more complex than expected.

## Introduction

Fungal invasions represent one of the most difficult infectious diseases to cure nowadays, causing worldwide more than 1.6 million deaths per year. People are constantly exposed to fungi, which are controlled in a first line of defense by the innate immune system through the phagocytosis of inhaled spores by macrophages and especially through neutrophils. Nevertheless, fungi can cause diseases such as airway allergy, bronchitis in healthy patients while it may cause deadly invasive infections in immunocompromised patients (1, 2).

The simpler immune system of the genetic model organism *Drosophila melanogaster* makes the analysis of host-pathogen interactions during infections easier to investigate as it lacks the adaptive immunity arm found in higher vertebrates. The host defense against pathogens in *Drosophila* mainly encompasses three major arms: melanization, the cellular response (essentially phagocytosis), and the humoral immune responses (*Toll* and Immune deficiency (*IMD*) NF-κB pathways). The *IMD* pathway is required to fight off Gram-negative bacterial infections whereas the *Drosophila* defenses against some Gram-positive bacteria and fungi mostly rely on the Toll pathway. Following the sensing of cell wall compounds or of the proteolytic activity of secreted microbial virulence factors in the hemolymph by circulating receptors, host proteolytic cascades will lead to the production of a mature Toll ligand, which will trigger an intracellular signaling pathway that activates the NF-κB-like transcription factors Dorsal or Dorsal immune-related factor (DIF) (3–5). DIF mediates *Toll* pathway function in innate immunity in adult flies while it is redundant with Dorsal in larvae (6–8). The *Toll* pathway regulates the expression of tens of genes, including those encoding antimicrobial peptides such as Drosomycin as well as those coding for the less characterized *Drosophila*-induced Immune Molecules (DIMs)/Bomanins, effectors that may act in conjunction with as yet unidentified cofactors (9–12)

*Aspergillus fumigatus* is the fungus that was initially used to demonstrate that Toll pathway mutants are sensitive to fungal infections (13). Given its medical relevance, we have implemented an unbiased genetic screen in which we monitor the survival of mutant *Drosophila* lines to injected conidia of this fungus. To this end, we are using transgenic lines that express miRNAs designed to target specific genes under the control of a Gal4 driver expressed ubiquitously (14), as well as other RNAi lines (15). To bypass the developmental lethality potentially caused by the down-regulated expression of the targeted gene, the RNAi transgene is expressed only at the adult stage using the Gal80 thermosensitive system (16). In the screen, we found that flies knocked down by RNAi targeting either of two Mediator complex subunit genes (*Med8*, *Med 31)* by RNAi transgene at the adult stage succumb to *A. fumigatus* infection.

The Mediator complex, evolutionarily conserved from yeast to plants, invertebrates, and mammals, consists of a multiprotein complex (25 subunits in yeast and 33 subunits in mammals) which plays an essential role for the transcription of almost all genes transcribed by RNA polymerase B (Pol B) (17–19). The Mediator complex is composed of a central module and a CDK8 kinase module (CKM). The central module consists of three complexes of distinct subunits known as the head, the middle, which together form the core module, and the tail parts. The central module associates with the CKM, which contains four subunits, and co-activates the transcription of target genes, yet does not appear to be fully essential for Mediator function. The Mediator core complex serves as a functional bridge connecting a variety of transcription activators bound to enhancer regions to the transcriptional machinery at the basal promoter, which includes Pol B and general transcription factors, to initiate gene transcription. The first step of gene transcription is the binding of transcription factors to the enhancer regions. Then, there is a subsequent recruitment of the Mediator complex to the enhancer regions by its interaction(s) with transcription factors through the tail and middle modules. Finally, general transcription factors and Pol B are recruited through the core module to form the preinitiation complex on the core promoter of the target gene (20). Gene transcription is then initiated, a process facilitated by the Mediator complex. In fact, Mediator complex can contact hundreds of transcription activators through its different subunits, generally the tail ones, and transduces the signals from specific transcription factors under different conditions (21). All subunits of the Mediator complex are recruited to the enhancer region; the CDK8 kinase module is transiently dissociated from the complex during the interaction with core promoters, however, its exact role is still not clear (20). Besides its role in the initiation of transcription, other studies have revealed that the Mediator complex also play roles at some other stages of transcription such as elongation, termination, processing of mRNA, and in epigenetic regulation as well as noncoding RNA activation (22).

Even though Mediator is required for the transcription of nearly all Pol B transcripts, half of its subunits appear to have specific functions. In *Drosophila*, most studies have focused on the role of the Mediator complex during development, *e.g (*23, 24*)*. A previous study revealed that one of the *Drosophila* Mediator complex subunits, dTRAP80 (a homologue of the head module component Med17), is required for DIF-dependent transcriptional activation of the *Drosomycin* gene in cultured cells and *in vitro*-translated Med17 has been shown to physically interact with both Dorsal and DIF by GST pull down (25). As stated above, two independent hits of our genetic screen are *Med8* and *Med31*. Med31 but not Med8 may bind to DIF and Dorsal and both have been reported to bind to Med17 in a large-scale effort to map protein-protein interactions in *Drosophila* by coaffinity purification of protein complexes (26). Whereas Med17 and Med8 both belong to the head module, Med31 belongs to the middle module, yet interacts, possibly indirectly, with several head module subunits besides Med17: Med6, Med11, Med18, and Med20 of the head module, and also Med7 from the middle module and Med14. Med8 appears to associate with subunits from all three central modules, including the Med14 scaffolding subunit, Med6, Med11, Med 17, and Med18 of the head module, Med4, Med7, Med10 of the middle module and Med15, Med16, Med23, Med25, Med27, and Med30 of the tail module (27). We therefore hypothesized that *Med31*, and possibly *Med8*, may play a role in *Drosophila* host defense against *A. fumigatus* infection through the facilitation of transcriptional activation mediated by DIF. We have extensively characterized the *Med31 A. fumigatus* susceptibility phenotype and also investigated the function of *Med31* in host defense against other pathogens including the dimorphic or monomorphic yeasts *Candida albicans* or *Candida glabrata*, the entomopathogenic fungus *Metarhizium robertsii*, the Gram-positive bacterium *Enterococcus faecalis* and the Gram-negative bacterium *Erwinia carotovora carotovora 15* (*Ecc15*). The Toll pathway is required for host defense against all these pathogens, except the last one. We further studied the role of most other identified Mediator subunits in *Drosophila* host defense, with a special emphasis on Med17, the subunit reported to directly bind to DIF. Our results delineate an unexpectedly complex picture of the Mediator complex in host defense that does not fit with it acting solely through the DIF transcription factor.

## Materials and Methods

### Fly Strains and Husbandry

Flies were raised at 25°C, 60% humidity with 12 h of light/dark cycle. The flies were fed with a semi-solid medium which consists of 7.8% w/v of corn flour, 6.3% w/v of glucose, 3.2% w/v of yeast dry powder, 0.9% w/v of agar, 0.2% w/v of sorbitol (except for *A. fumigatus* infected flies because *A. fumigatus* is sensitive to the sorbitol preservative), 0.07% w/v of CaCl2, 3.2% w/v of sucrose, 0.15% w/v of p-Hydroxybenzoic Acid Methyl Ester and water.

The TRiP RNAi lines were obtained from the *TsingHua Fly Center* (THFC), the GD RNAi lines come from the *Shanghai Institute for Biological Sciences* (originally from the Vienna Drosophila Research Center, Austria). The insert was checked by sequencing for each line. UAS-mCherry-sh (BDSC_35785) and VDRC_60000 were used as controls for TRiP lines and GD lines, respectively. **Supplementary Table 1** lists the RNAi lines used in this study. Males from the RNAi lines or their controls were crossed with Ubi-Gal4, tub-Gal80^ts^ virgins. Crosses were set-up at 25°C for three days to ensure an efficient fertilization of females by males. Adults were then transferred to another tube while the tube containing eggs was moved to 18°C, in order to keep the inhibition of Gal4 by Gal80^ts^ and bypass developmental lethality.

Soon after the F1 progeny hatched, flies were shifted at 29°C to inhibit Gal80^ts^ and activate Gal4 to initiate the transgene expression. Flies were kept at 29°C for 5 days to ensure the down-regulation of the genes of interest prior to immune challenge.

### Microbial Strains and Growth Conditions

The RFP-labeled wild-type *Aspergillus fumigatus* strain was a kind gift from Drs. Anne Beauvais and Jean-Paul Latge (Institut Pasteur, Paris). *A. fumigatus* was cultured on potato dextrose agar (PDA) medium plates supplemented with 0.1 g/l chloramphenicol (Huankai Microbio Tech, China) in a tissue culture incubator under 5% CO_2_ at 29°C. *Metarhizium robertsii* (ARSEF 2575) was grown on PDA plates from BD Company, USA (#213400) at 25°C for 7 to 14 days. Of note, we did not use the same PDA plates for *A. fumigatus* and *M. robertsii* because the PDA used for *A. fumigatus* contains chloramphenicol, which affects the growth of *M. robertsii*.

*Candida albicans* (CAF 2.1) and *Candida glabrata* (28) were cultured on Yeast extract Peptone Dextrose (YPD) Agar plates for two days at 29°C, from which one colony was plated on a new plate again for two days at 29°C, and then this plate was kept at 25°C for infections for four weeks.

*E. faecalis* (OG1RF) and the *Pectobacterium carotovorum* strain *Erwinia carotovora carotovora* (*Ecc15*) were cultured in Luria-Bertani (LB) agar plates overnight at 37°C. A single colony was inoculated in LB liquid medium at 37°C and overnight shaking.

### Microbial Preparation and Infection Experiments

Prior to *A. fumigatus* injection or *M. robertsii* natural infection, flies were raised on 100 mM sucrose for two days to eliminate sorbitol (an antifungal compound present in the fly food) from the flies. Prior to *M. robertsii* injection, flies were raised on regular food. *M. robertsii* and *A. fumigatus* conidia were collected from the surface of the PDA plate by adding three ml of either PBST (PBS containing 0.01% Tween-20) for injections, or sterilized deionized demineralized water containing 0.01% Tween-20 for natural infections. The concentration of the conidia was counted using a hemocytometer and then adjusted to the adequate working concentration. The working concentrations were 5x10^7^ *A. fumigatus* conidia/ml and 1x10^7^ *M. robertsii* conidia/ml in PBST for injections, and 5x10^4^ conidia/ml in water containing 0.01% Tween-20 for *M. robertsii* natural infection.

For *M. robertsii* natural infection, anesthetized flies were incubated into the conidia solution and shaken for 30 s, before being dried on Millipore AP1003700 filter paper adapted to a vacuum pump. *M. robertsii*-naturally infected flies were then raised on a vial containing a filter paper with 100 mM sucrose. *A. fumigatus*-injected flies were kept on food without sorbitol whereas *M. robertsii*-injected flies were fed regular food.

Overnight cultures of *E. faecalis* and *Ecc15* were centrifuged at 3,500 g, 4°C for 10 min. The pellet was washed twice in PBS. *E. faecalis* and *Ecc15* were prepared in PBS at working concentrations of OD600 = 0.1 and 1, respectively.

Injection of *A. fumigatus*, *M. robertsii*, *E. faecalis* or *Ecc15* was performed by injecting 4.6 nL of working solutions, or the same volume of PBS as a control, into flies by using a microinjector (Nanoject, Drummond) and appropriate capillaries. Infection of *C. albicans* or *C. glabrata* was performed by pricking through a sharpened tungsten needle dipped into a single colony directly taken on plates (28).

### Survival Assays

The survival assays were performed with 15–25 females per tube, in triplicates. Infected flies were incubated at 29°C with 60% humidity. Log-Rank statistical tests were performed with GraphPad Prism 6. Experiments were performed at least twice, except for the following experiments that were performed only once: *M. robertsii* natural infections: *Med6*, *Med8*, *Med11*, *Med22*, *Med24*; *C. glabrata* infections: *Med12*, *Med14*, *Med21*; *E. faecalis* infections: *Med11*, *Med12*; *Ecc15* infections: *Med11*, *Med12*.

### Detection of AMP Expression Level After Infection

RNA was extracted from four flies per sample in triplicates or quadruplicates. Flies were homogenized with 1 ml of Trizol reagent (Thermo Fisher Scientific, #15596018) in a microfuge tube using a pestle and the RNA extraction was performed according to the manufacturer instructions. Two hundred μL of chloroform was added to the samples, which were vortexed and incubated for 5 min at room temperature. Then, samples were centrifuged at 13,000 g at 4°C for 10 min. The water phase at the top of the samples was collected and mixed with the same volume of isopropanol and vortexed again. Samples were centrifuged at 13,000 g at 4°C for 10 min. The pellet was washed with a solution of 75 % ethanol in water and air dried. Then, RNAs were re-suspended in 35 μl of RNase-free water. The quality and the concentration of the total RNA were measured using a Nanodrop 2000 (Thermo Fisher Scientific). The reverse transcription of 800 ng of total RNA was made with a cDNA synthesis kit (TransGene Biotech, #AT-341), Quantification of the target gene expression level was performed by quantitative Polymerase Chain Reaction (qPCR) with the SYBR Green Supermix kit (Vazyme Biotech, Q311-02). The relative gene expression was normalized to the expression level of the housekeeping gene *Rpl32*, which encodes ribosomal protein 49. Digital PCR was performed on cDNAs at 1 ng/µL as described (29). The list of primers used for this study is found in **Table S2**.

Wild-type flies pricked with concentrated cultures of *Micrococcus luteus* were used as positive controls for *Drosomycin* and *DIM1*, or *Escherichia coli* for *Diptericin* expression (30). The ΔΔCt method was used to normalize the values as following: we gave a value of 1 for the expression level obtained for *Drosomycin* and *DIM1* 24 h after infection with *M. luteus* and for the expression level obtained for *Diptericin* 6 h after infection with *E. coli*.

### Western Blot

Four hours after a challenge with *M. luteus*, the hemolymph from 20 flies was collected into 40 μl of PBS containing Protease Inhibitor Cocktail (Thermo Fisher Scientific) by centrifugation (3,500 g, 30min, 4°C) after cutting the tip of the abdomen (28). The protein concentration of the sample was measured by using a BCA kit (Beyotime Biotechnology). Samples were mixed with SDS-PAGE loading buffer (Beyotime Biotechnology) and boiled for 5 min. A SDS-PAGE electrophoresis gel was performed using 20 μg of protein. Proteins were transferred to a polyvinylidene fluoride (PVDF) membrane (0.22 μm).

The nitrocellulose membrane with transferred proteins was blocked in PBST with 5% BSA at room temperature for 1 h. The membranes were incubated in a 1:10,000 rabbit anti-PPO1 primary antibody solution (a kind gift from Erjun Ling, Shanghai) in PBST with BSA overnight at 4°C (31). The membranes were washed and incubated in a secondary anti-rabbit HRP antibody (1:10000) for 1 h at room temperature.

### Phagocytosis Assay

Three to seven days old adult females were injected with 69 nL of latex beads (Thermo Fisher Scientific), 16% w/v (re-suspended in PBS and sonicated prior injection). Control flies were injected with the same volume of PBS. Twenty-four hours post-injection, flies were injected with 69 nL pHrodo™ Red E. coli BioParticles™ Conjugate (Thermo Fisher Scientific, P35361). The phagocytic activity was observed under a fluorescence microscope (Zeiss Imager.M2) after 30 min. The red fluorescence was quantified in fields of same size. Ten flies of each line were scored in each experiment and three independent experiments were performed.

### Microbial Load Counts

*A. fumigatus* and *C. glabrata* microbial loads after infection were counted after plating a homogenate of single whole flies. Each single fly was transferred into a 1.5 ml Eppendorf tube containing 50 μl of PBST and smashed by a tissue homogenizer. After a few seconds of centrifugation, the entire homogenate product was plated on PDA, and incubated at 29°C. Colony forming units (CFUs) were counted after 2–3 days of incubation. In the case of *C. glabrata*, a 1:100 or 1:1,000 dilution of the homogenate product was plated depending on the time after infection.

*E. faecalis* loads were counted on hemolymph collected from single flies. A series of dilution was performed from 1 to 1:10^8^ in PBS. Ten μl of each dilution was plated in duplicate on LB agar and incubated at 37°C. Colony forming units (CFUs) were counted the next day.

### Statistical Analysis

All statistical analyses were performed by using GraphPad Prism 7. The Mann-Whitney or Kruskall-Wallis tests was used for the statistical analysis of all the data except survival experiments. Survival curves were plotted and analyzed by Log-Rank test (Kaplan-Meier method).

## Results

### *Med31* RNAi Flies are Susceptible to Injected *Aspergillus fumigatus* Conidia

**Figure 1:**
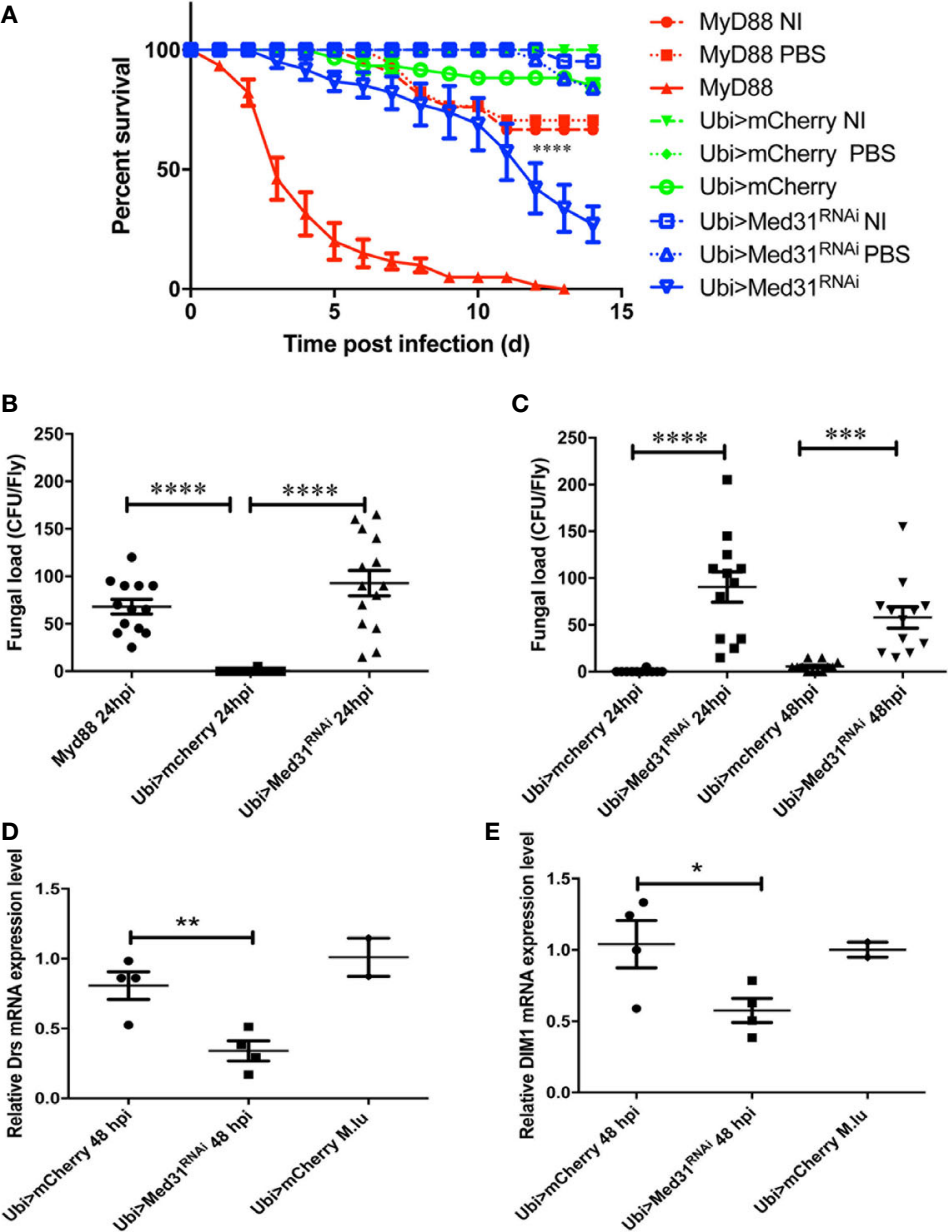
Med31 RNA flies are susceptible to *A. fumigatus* infection. **(A)** Survival of Drosophila after *A. fumigatus* infection. *MyD88* is the positive control line (red), Ubi>*mCherry* RNAi line is the wild type control line (green) and Ubi>*Med31* RNAi line is the experimental line (blue). Each infected line has a non-infected (NI, dashed lines) and PBS-injected control (dotted lines). *Med31* RNAi flies succumbed faster to infection than the wild type controls. Infected *Med31* RNAi flies *vs.* infected *mCherry* RNAi flies: log-rank test, ****P < 0.0001. The survival curves are representative of 10 independent experiments. **(B, C)** Fungal load of whole single flies after 24 and 48 h of infection. Each dot represents a single fly. The fungal load was higher in *MyD88* and *Med31* RNAi flies than in control flies at 24 h post infection. It did not increase in *Med31* RNAi flies at 48 h post infection compared to 24 h. ****P < 0.0001. ***P < 0.001. **(D, E)** Expression levels of *Drosomycin* **(D)** and *DIM1* **(E)** at 48 h post infection, normalized to *Rpl32* (RP49 protein coding gene) housekeeping gene expression. The expression of control flies challenged with the nonpathogenic Gram-positive bacterium *Micrococcus luteus* is given for reference. Each dot represents one sample containing four flies. *Med31* RNAi flies displayed decreased *Drosomycin* and *DIM1* expression levels at 48 h post infection. *P < 0.05. Mean ± SEM are indicated **(B–E)**.

We have established that as little as five *A. fumigatus* conidia injected on average per fly are sufficient to kill *MyD88*-immunodeficient flies and are routinely injecting 250 conidia per fly (Xu *et al*., *in preparation*). As shown in **Figure 1A**, this dose rapidly kills *MyD88* whereas only a moderate proportion of *mCherry* RNAi control flies succumbed to this challenge in most experiments. *Med31* RNAi flies displayed a high sensitivity to injected *A. fumigatus* in ten independent experiments, which was however not as pronounced as for *MyD88* flies. We confirmed this result using two other independent RNAi lines and also checked by classical (not shown) and digital RT-qPCR that all three RNAi lines effectively decreased the steady-state levels of MED31 transcripts (**Figures S1A–C**). Next, we measured the fungal load to determine whether it increases in the mutant background, as has been reported for immuno-deficient flies challenged with pathogens (28, 32). At 24 h after infection, the titer was somewhat higher in *MyD88* and *Med31* RNAi flies than in *mCherry* RNAi control flies (**Figure 1B**). However, the fungal burden did not increase at 48 h (**Figure 1C**). We next monitored the induction of the Toll pathway using the expression level of two of its target genes, *Drosomycin* and *BomS1*/*DIM1* (33). The injection of 250 A*. fumigatus* conidia mildly induced the expression of these two genes, which was reduced in *Med31* RNAi flies (**Figures 1D, E**). Thus, *Med31* is required for the full transcriptional induction of *Drosomycin*, likely by recruiting Pol B to the *Drosomycin* and *DIM1* promoters bound by DIF.

### *Med31* RNAi Flies Display a Moderate Sensitivity to *Enterococcus faecalis* Infection

**Figure 2:**
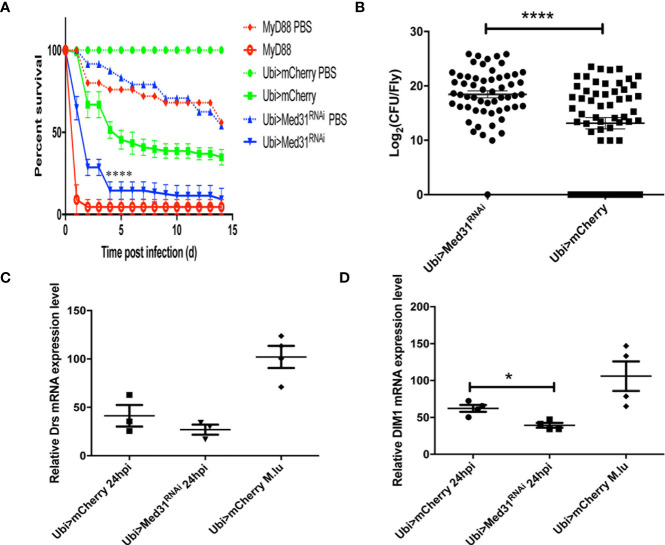
Med31 RNAi flies are susceptible to *E. faecalis* infection. **(A)** Survival of Drosophila after *E. faecalis* infection. *MyD88* was used as a positive control line (red), Ubi>*mCherry* RNAi line represented the wild type control line (green) and the Ubi >*Med31* RNAi line survival is shown in blue. For each infected line a PBS-injected control was also performed (dotted lines). *Med31* RNAi flies succumbed faster to infection than the wild type controls. Infected *Med31* RNAi flies *vs.* infected *mCherry* RNAi flies: log-rank test, ****P < 0.0001. The survival curves are representative of nine independent experiments. **(B)** Bacterial load in the hemolymph after 24 h of infection. Each dot represents the burden of a single fly. The bacterial load in the *Med31* RNAi flies was higher than the controls at 24 h post infection. ***P < 0.001. **(C, D)** Expression level of *Drosomycin* **(C)** and *DIM1* **(D)** normalized to the *Rpl32* house keeping gene 48 h post infection. Each dot represents one sample containing four flies. Septic infection with *M. luteus* was a positive control for *Drosomycin* and *DIM1* expression. *Drosomycin* expression level in *Med31* RNAi flies was not significantly different from control flies **(C)** but *DIM1* expression level was significantly decreased at 24 h post infection. *P < 0.05. Mean ± SEM are indicated **(B–D)**.

When challenged with *E. faecalis*, *Med31* RNAi flies displayed a sensitivity to this infection that was intermediate between those of *MyD88* and wild-type control flies in seven out of nine experiments (**Figure 2A**), whereas they behaved almost like wild-type flies in the two other experiments. To corroborate these results, we measured the bacterial load and found that it was increased on average 32-fold in the *Med31* RNAi flies with respect to *mCherry* RNAi control flies (**Figure 2B**). Unexpectedly, we did not find a significant decrease in *Drosomycin* induction by *E. faecalis* challenge in three independent experiments, although the mean induction of *Drosomycin* was somewhat decreased when compared to controls (**Figure 2C**); in a digital RTqPCR experiment, we indeed found a significant difference. However, there was a significant difference when monitoring another read-out of Toll pathway activation, *BomS1/DIM1* transcript levels (**Figure 2D**), which was confirmed by digital RTqPCR. The reduction was however modest. Thus, *Med31* RNAi flies appear to have reduced host defenses against two pathogens known to be effectively killing Toll pathway-deficient flies.

### Test of *Med31* RNAi Flies in Further Infection Models

**Figure 3:**
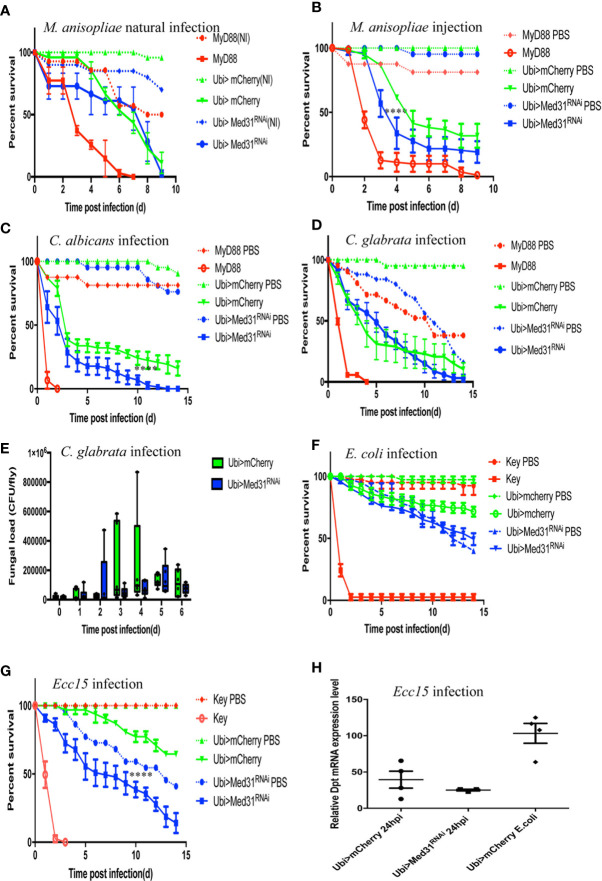
Susceptibility of *Med31* RNAi flies to other pathogens. Survival of *Med31* RNAi flies challenged with other pathogens. *MyD88* represents the positive control line (red) for flies infected with *M. anisopliae*, *C. albicans*, *C. glabrata*, and *E. faecalis*; *kenny*, a member of the IMD pathway, is the positive control line (red) for fly lines infected with *Ecc15* or *E. coli*, Ubi>*mCherry* RNAi line corresponds to the wild type control line (green) and Ubi>*Med31* RNAi line (blue). For each infected line a PBS-injected control was also performed (dotted lines). **(A)** Survival of *Med31* RNAi flies after *M. robertsii* natural infection. The survival curve is representative of nine independent experiments. **(B)** Survival of *Med31* RNAi flies following *M. robertsii* conidia injection. Infected *Med31* RNAi flies *vs.* infected *mCherry* RNAi flies: log-rank test, ****P < 0.0001. The survival curves are representative of five independent experiments. **(C)** Survival of *Med31* RNAi flies following *C. albicans* infection. Infected *Med31* RNAi flies *vs.* infected mCherry RNAi flies: log-rank test, ****P < 0.001. The survival curves are representative only of two out of four independent experiments, the other two not displaying any difference between *Med31* RNAi and control flies. **(D, E)** Survival of *Med31* RNAi flies and the fungal load following *C. glabrata* infection. The survival curves are representative of nine independent experiments. **(F)** Survival of *Med31* RNAi flies following *E. coli* infection. The survival curves are representative of two independent experiments. **(G)** Survival of *Med31* RNAi flies after *Ecc15* challenge. The survival curves are representative of nine experiments. **(H)** *Diptericin* expression level normalized to the *Rpl32* house keeping gene at 24 h post infection with *Ecc15*. Each dot represents one sample containing four flies. Septic infection with *E.coli* was a positive control for *Diptericin* expression. Mean ± SEM are indicated, except for 3E where the median is displayed.

The Toll pathway has been reported to play an essential role in host defense against entomopathogenic fungi such as *Metarhizum robertsii* and pathogenic yeasts such as *Candida albicans* and *C. glabrata* (4, 28, 34, 35). Entomopathogenic fungi invade the host body cavity upon the deposition of spores on the cuticle of the insect (“natural” infection model) or can be artificially directly injected inside the fly (septic injury model mimicking a wound). Interestingly, we found in nine “natural” infection experiments that *Med31* RNAi flies behaved as wild-type control flies in this infection paradigm (**Figure 3A**) whereas they displayed a moderate but reproducible susceptibility to injected *M. robertsii* in five experiments (**Figure 3B**). With respect to pathogenic yeasts, *Med31* RNAi flies displayed a weak susceptibility to *C. albicans* in two out of four experiments (**Figure 3C**) while it was not sensitive to *C. glabrata* in six out of nine survival experiments (**Figure 3D**). We found that the *C. glabrata* burden was not differing between *mCherry* RNAi and *Med31* RNAi flies during the course of the infection (**Figure 3E**). Thus, *Med31* does not appear to be required to the same extent in host defense against microbial infections depending on the pathogen, even though all of these microbes are controlled, at least to some degree, by the Toll pathway. We also checked whether *Med31* affects the host defense against Gram-negative pathogens. The weakly pathogenic *Escherichia coli* did not kill *Med31* RNAi flies more efficiently than a PBS-injection control in two independent experiments (**Figure 3F**). We found that *Med31* RNAi flies were also insensitive to *Ecc15* challenge in seven out of 11 experiments (**Figure 3G**). We also checked whether IMD pathway signaling was affected in *Med31* RNAi flies by measuring the steady-state transcript levels of *Diptericin*. No significant difference was recorded (**Figure 3H**). In conclusion, the requirement for full *Med31* function in host defense against bacterial or fungal infection varies according to the pathogen and the unique suite of host defenses engaged in each case. As *Dif* has been reported to be required in host defense against *M. robertsii* in the “natural infection” model, the lack of a requirement for *Med31* in this infection is unexpected given its involvement in the host defense against *A. fumigatus*, *E. faecalis*, and injected *M. robertsii* conidia. We cannot however exclude the possibility that another Mediator subunit mediates an interaction with DIF in the other infections that are not modulated through Med31.

### *Med31* Does Not Appear to be Required for the Melanization nor for the Cellular Immune Response

**Figure 4:**
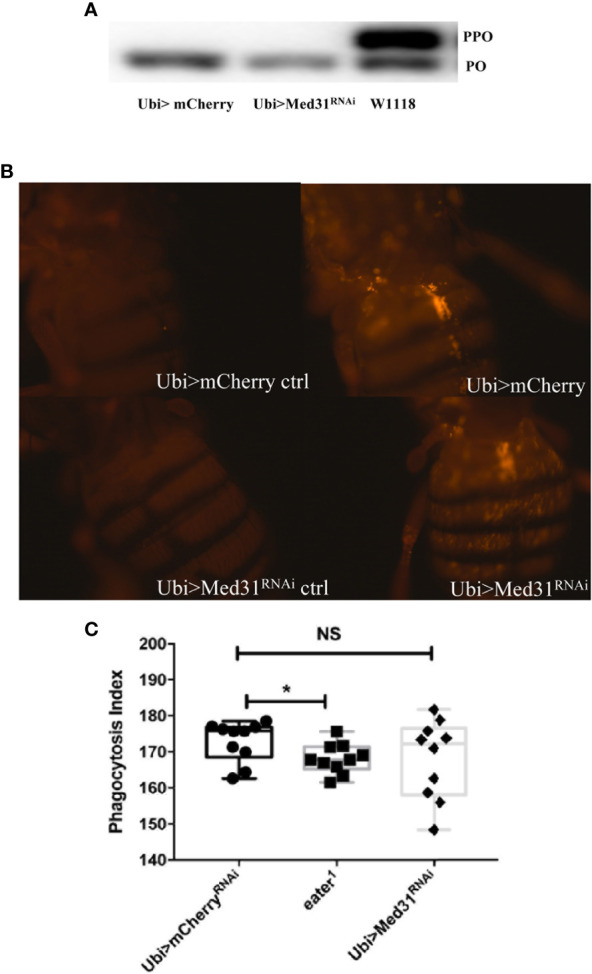
Melanization and phagocytosis are not affected in *Med31* RNAi flies. **(A)** prophenol oxidase (PPO) cleavage four hours after a *M. luteus* challenge was detected by Western blot analysis. The cleavage of PPO into phenol oxidase (PO) is shown in the picture. PPO cleavage into PO was total for both *Med31* RNAi and *mCherry* RNAi control flies, while that induced in another wild-type control, w1118, was only partial. This blot is representative of three out of four independent experiments. The phagocytic activity of flies was detected using injected pH-RODO-labelled *E. coli*, which become fluorescent when internalized into mature phagosomes. **(B)** Flies were injected with latex beads as a phagocytosis-deficient control (left panels). The phagocytic activity of *Med31* RNAi flies was not altered compared to the *mCherry* RNAi control flies (right panels). **(C)** Quantification of the fluorescence emitted by internalized bacteria. The *eater^1^* phagocytosis-deficient mutant flies represent a positive control. There was no significant difference between *Med31* RNAi flies and the *mCherry* RNAi control flies. Each dot represents the fluorescence measured in a single fly. Three independent experiments were performed. *P < 0.05. Median ± SEM are indicated. NS: not significant.

We have tested whether *Med31* plays a role in two other host defenses, melanization and phagocytosis. We did not notice any alteration of the melanin plug formed at the wounding site in *Med31* RNAi flies. We further tested at the molecular level whether the proteolytic processing of prophenol oxidase into mature phenol oxidase was impaired in these flies, as this represents a key step in the melanization response. We found in four out of five experiments that prophenol oxidase was equally or better cleaved in the mutant flies as compared to wild-type controls whereas in one experiment a minor unprocessed form remained while the control was fully cleaved (**Figure 4A**). We conclude that *Med31* does not influence melanization after a septic injury.

We next checked whether the basal phagocytic machinery was functional in *Med31* RNAi flies by injecting pH-rodo-labeled *E. coli* that emit red fluorescence when placed in an acidic environment such as that encountered in mature phagolysosomal vesicles. **Figures 4B–C** show that the uptake of these particles by hemocytes located on the fly dorsal vessel was not dramatically altered when *Med31* expression was ubiquitously knocked-down.

### A Mini-Screen to Identify Other Med Subunits Involved in Host Defense Against *Aspergillus fumigatus*

To obtain a better understanding of the role of the Mediator complex in host defense in the *Drosophila* model, we decided to test the available RNAi lines targeting the genes encoding other subunits of this complex. A limitation of the RNAi approach is that the efficiency of interference may be varying. As the Mediator complex plays an essential role in development, we reasoned that expressing the RNAi transgene throughout development should severely alter the proportion of adult flies hatching from a cross between the RNAi line and a ubi-Gal4 driver line. This strategy should therefore allow us to validate the efficiency of the RNAi lines in blocking their targets. Indeed, we found that this was the case for most tested RNAi lines, *Med4*, *Med9*, *Med10*, *Med18*, and *CDK8* excepted (**Table 1**). We thus directly measured the efficiency of most of the RNAi lines that did not pass this test by RTqPCR (not shown) and digital RTqPCR (**Figure S2**). *Med18* excepted, the tested lines displayed a strong decrease of the targeted transcripts, suggesting that the corresponding Mediator subunits (Med4, Med9, and Med10) may not play an essential role during development.

**Figure 5:**
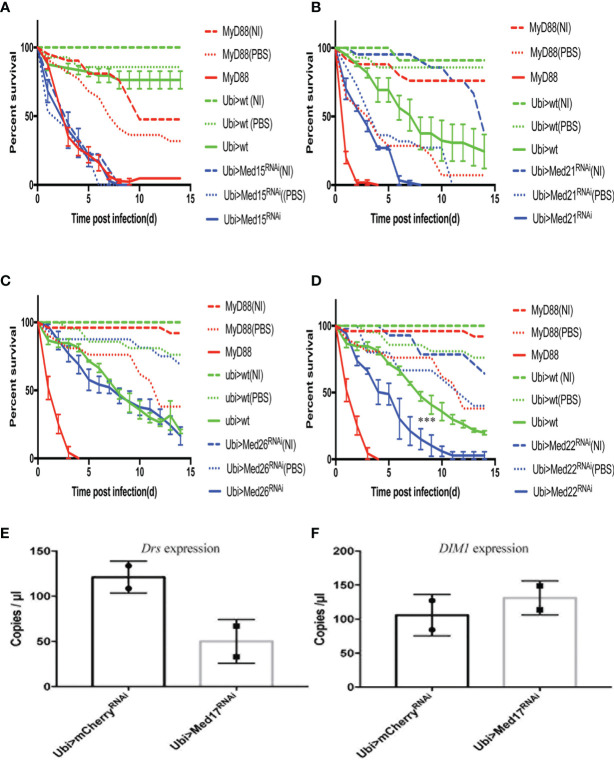
The distinct survival phenotypic categories for other Mediator complex subunits RNAi flies challenged with *A. fumigatus*. Survival of RNAi lines targeting genes encoding other Mediator complex subunits after *A. fumigatus* infection. *MyD88* represents the positive control line (red), Ubi>*mCherry* RNAi line the wild type control line (green), and Ubi> RNAi line of Mediator complex subunits is shown as a blue line. Each infected line has a non-infected (NI, dashed lines) and a PBS-injected control (dotted lines). **(A)** Example of a lethal phenotype, uninfected *Med15* RNAi flies succumbed at the same rate as challenged flies. **(B)** Example of a wound-sensitive phenotype, *Med21* RNAi flies were sensitive to the injection of PBS. **(C)** Example of an absence of sensitivity phenotype, *Med26* RNAi flies did not show any enhanced sensitivity to infections. **(D)** Example of sensitivity to *A. fumigatus* infection. *Med17* RNAi flies displayed a *Med31*-like phenotype. Infected *Med17* RNAi flies *vs.* infected *mCherry* flies: log-rank test, ****P < 0.001. **(E, F)** Expression level of *Drosomycin* **(E)** and *DIM1* **(F)** at 48 h post *A. fumigatus* infection measured by digital RTqPCR. Each dot represents one sample containing five flies. Mean ± SEM are indicated.

We have performed survival analysis on 30 RNAi lines after *A. fumigatus* challenge by expressing the RNAi transgene only at the adult stage. Eight lines yielded a lethal phenotype, that is, uninfected flies succumbed at the same rate as challenged flies (**Table 2**, **Figure 5A**). Two lines, *Med21* and *Med27*, displayed a heightened sensitivity to the control injection of PBS, indicating that they are highly-wound sensitive (**Table 2**, **Figure 5B**). In both cases, it is thus not possible to assess whether these subunits are specifically involved in host defense. We did not find any enhanced sensitivity to fungal infections for 12 lines (Table 2, **Figure 5C**). Thus, 10 subunits (Med4, Med9, Med10, Med19, Med20, Med23, Med 24, Med25, Med26, and CycC) do not appear to play an essential role in host defense against *A. fumigatus* since the corresponding RNAi transgene is clearly functional. Finally, seven lines displayed a *Med31*-like phenotype, although a significant proportion of PBS-injected controls succumbed in the case of *Med 11* (**Table 2**, **Figure 5D**, **Figure S3**).

**Table 1:** Validation of Med RNAi flies lethality at 29°C.

RNAi strain	Pupae	Hatching flies (%)	RNAi strain	Pupae	Hatching flies (%)
mCherry	51	51 (100%)	Med19	27	0
Med1	0	0	Med20	20	0
#Med4	30	25 (83.33%) 10 (33.33% homozygous) 15 (50% heterozygous)	Med21	0	0
Med6	0	0	Med22	0	0
Med7	0	0	#Med23	0	0
Med8	0	0	Med24	0	0
Med9	4	4 (100%)	Med25	0	0
#Med10	12	100% 4 (33.33% homozygous) 8 (66.67% heterozygous)	Med26	0	0
Med11	0	0	Med27	0	0
Med12	0	0	Med28	0	0
Med13	0	0	Med29	0	0
Med14	0	0	#Med30	0	0
Med15	0	0	Med31	0	0
Med16	8	0	Cyc C	14	0
Med17	0	0	CDK8	14	12 (85.71%)
Med18	12	10 (83.3%)			

Flies were crossed with Ubi-Gal4,tub-Gal80^ts^ virgins (two males and four females per tube) at 29°C, and parents were discarded five days post crossing. The offspring was raised at 29°C, and their viability was assessed.

#Some UAS-RNAi lines are heterozygous (no homozygous flies found). Therefore, in the progeny of the crosses done with Gal4 lines, there are flies carrying a balancer chromosome and not the UAS-RNAi transgene: these can be identified only at the adult stage, thanks to their genetic markers.

**Table 2:** Other Med subunits in host defense against *A. fumigatus*.

Phenotype	MED subunits
With phenotype	Med 6, Med 8, Med 11, Med14, Med17, Med22, Med30
Lethal	Med1, Med7, Med12, Med13, Med15, Med16, Med28, Med29
Wound sensitive	Med21, Med 27
No phenotype	Med4, Med9, Med10, *Med18*, Med19, Med20, Med23, Med24, Med25, Med 26, Cyc C, *CDK8*

The subunits indicated in italics may not be efficiently silenced by RNAi and the absence of a phenotype may just reflect this technical problem, thereby preventing a solid conclusion to be drawn.

### The *Med17* Subunit RNAi Mutants Display a Sensitivity Only to *Aspergillus fumigatus* and *Enterococcus faecalis* Infections

**Figure 6:**
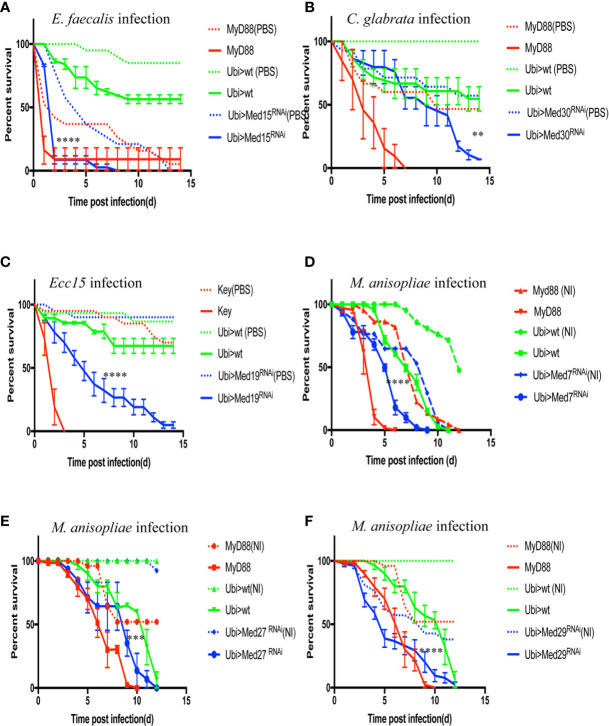
Survival of RNAi flies targeting different Mediator complex subunits after challenge with various pathogens. Survival of other Mediator complex subunits RNAi lines after challenges with either *E. faecalis*, *C. glabrata*, *Ecc15*, or *M. robertsii. MyD88* was used as positive control line (red) for *M. robertsii*, *C. glabrata* or *E. faecalis* infection, and *key* was the positive control line (red) for lines infected with *Ecc15*. Ubi>*mCherry* RNAi line was the wild type control line (green) and Ubi> RNAi line of Mediator complex subunits is displayed in blue. **(A)** Survival curves of *Med17* RNAi flies, which were susceptible to *E. faecalis* infection. Infected *Med17* RNAi flies *vs.* infected *mCherry* RNAi flies: log-rank test, ****P < 0.0001. The survival curves are representative of six experiments. **(B)** *Med30* RNAi flies were susceptible to *C. glabrata* infection. Infected *Med30* RNAi flies *vs.* infected *mCherry* RNAi flies: log-rank test, **P < 0.01. The survival curves are representative of two experiments. **(C)** *Med19* RNAi flies were susceptible to *Ecc15* infection. Infected *Med19* RNAi flies *vs.* infected *mCherry* RNAi flies: log-rank test, ****P < 0.0001. The survival curves are representative of two experiments. **(D–F)** *Med7*, *Med27*, and *Med29* RNAi displayed an enhanced sensitivity to *M. robertsii “*natural*”* infection. Infected *Med7* RNAi flies *vs.* infected *mCherry* RNAi flies: log-rank test, ****P < 0.0001. The survival curves are representative of three experiments. Infected *Med27* RNAi flies *vs.* infected *mCherry* RNAi flies: log-rank test, ***P < 0.001. The survival curves are representative of two experiments. Infected *Med29* RNAi flies *vs.* infected *mCherry* flies: log-rank test, ****P < 0.0001. The survival curves are representative of two experiments. Please, note that the mock-infected flies for *Med7* and *Med29* succumbed also rapidly to the procedure, making it difficult to conclude unambiguously on the role of these two subunits in the host defense against *M. robertsii* “*natural*” infection. Error bars are SEM.

Among the lines that shared with the *Med31* RNAi line a sensitivity phenotype to *A. fumigatus*, the *Med17* RNAi KD line is of special interest since it targets the expression of Med17, a Mediator complex subunit that has been shown to bind to DIF *in vitro* and to be required for the Toll-dependent induction of *Drosomycin* expression in cell culture (25). A similar phenotype was observed with two further independent RNAi lines (**Figures S1D, E**) and all three KD lines nearly abolished *Med17* expression (**Figure S1F**). It was therefore interesting to determine whether the *Med17* RNAi line displayed the same palette of sensitivity to specific microbial infections as the *Med31* one. Whereas the *Med17* KD line indeed displayed an increased sensitivity to *E. faecalis* infections in all six performed experiments (**Figure 6A**), it however was as resistant as control flies to injected *M. robertsii* conidia in the six survival experiments (**Figure S4A**), unlike the *Med31* RNAi line. Finally, the *Med17* RNAi line was not sensitive to *C. glabrata* in five out of six experiments and not susceptible to *Ecc15* in six experiments (**Figures S4B, C**). Thus, even though Med31 and Med17 bind to each other in the Mediator complex, their disruption leads to related but not identical phenotypes. Interestingly, whereas *Drosomycin* induction by a *M. luteus* challenge was reduced in the *Med17* KD line as expected, it did not affect the induction of *BomS1/DIM1* transcripts (**Figures 5E, F**), even though the *BomS1* gene contains a canonical DIF binding site in its promoter (33). Also of note, the expression of some other AMP genes by an *E. coli* challenge, namely *Drosocin and CecropinA* but not *Diptericin*, appeared to be upregulated in the *Med17* RNAi line (**Figures S4 D–G**).

### Role of Other Med Subunits in Host Defense Against Additional Pathogens

We next tested the susceptibility of other available RNAi lines against other Med subunit-encoding genes to *E. faecalis* and found that six of them displayed an enhanced sensitivity to this pathogen (**Table 3**, **Figure S5**). We had initially found that *Med12, Med15*, and *Med28* RNAi flies succumbed at the same rate whether challenged or not in two experiments with *A. fumigatus*. In the case of *E. faecalis* infection, the PBS-injected control did not die as rapidly as previously, whereas the bacteria-challenged ones succumbed much faster (**Figure S5**). Indeed, the *E. faecalis* burden was higher than in controls for the *Med 15* and *Med28* RNAi flies, indicating that these lines are indeed susceptible to *E. faecalis* and not solely to the injection procedure. The important observation is that the *Med6* and *Med11* lines displayed a sensitivity phenotype after *A. fumigatus* but not after *E. faecalis* infection.

We further challenged these Mediator subunit RNAi lines with *C. glabrata* or *Ecc15*. Most of them tested negative except for *Med30* in the case of the pathogenic yeast (**Figure 6B**) and *Med19* for *Ecc15* that displayed intermediate sensitivity to these pathogens (**Figure 6C**, **Tables 3**, **4**).

Finally, we used the *M. robertsii* natural infection model to characterize the Mediator subunit subset and found that *Med7, Med27*, and *Med29* RNAi displayed an enhanced sensitivity to this challenge although it should be noted that mock-infected *Med7* and *Med29* RNAi flies also succumbed during the course of this experiment, albeit at a somewhat slower rate (**Figures 6D–F**). This observation for *Med 7* and *Med 29* is in keeping with the lethality observed in the infections series with *A. fumigatus* (**Table 4**). Whereas Med27 is clearly required for host defense against a natural *M. robertsii* infection, we cannot determine unambiguously whether it is also required in the host defense against *A. fumigatus* given its sensitivity to the wound.

**Table 3:** Other Med subunits in host defense against other pathogens.

Pathogens	Other MED subunits
*E. faecalis*	Med 8, Med12?, Med15, Med17, Med22, Med28, Med30
*Ecc15*	Med 19
*C. glabrata*	Med 30
*M. robertsii* *Natural infection*	Med7?, Med27, Med29?

The question marks indicate that a sensitivity to the infection procedure may significantly contribute to the phenotype.

**Table 4:** Summary of Med subunits in host defense against pathogens.

	*A. fumigatus*	*E. faecalis*	*M. robertsii* natural infection	*C. glabrata*	Ecc15	*M. robertsii* Injection	Lethality at 29°C	RNAi efficiency by dPCR
Med1	L	–	–	–	–	/	++	/
Med4	–	–	–	–	–	/	+/-	+
Med6	+	–	–	–	–	/	++	/
Med7	L	–	+?	–	–	/	++	/
Med8	+	+	–	–	–	/	++	/
Med9	–	–	–	–	–	/	+/-	+
Med10	–	–	–	–	–	/	+/-	+
Med11	+	–	–	–	–	/	++	/
Med12	L	+	/	–	–	/	++	/
Med13	L	–	–	–	–	/	++	/
Med14	+	/	–	–	/	/	++	/
Med15	L	+	–	–	–	/	++	/
Med16	L	–	–	–	–	/	+	/
Med17	+	+	–	–	–	–	++	+
***Med18***	–	–	–	–	–	/	+/-	+/-
Med19	–	–	–	–	+	/	+	/
Med20	–	–	–	–	–	/	+	+
Med21	-?	–	/	–	/	/	++	/
Med22	+	+	–	–	–	/	++	/
Med23	–	–	–	–	–	/	++	/
Med24	–	–	–	–	–	/	++	/
Med25	–	–	–	–	–	/	++	/
Med26	–	–	–	–	–	/	++	/
Med27	-?	–	+	–	–	/	++	/
Med28	L	+	–	–	–	/	++	/
Med29	L	–	+?	–	–	/	++	/
Med30	+	+	–	+	–	/	++	/
Med31	+	+	–	–	–	+	++	+
Cyc C	–	–	–	–	–	/	+	/
*CDK8*	–	–	–	–	–	/	+/-	/

The underlined Med subunits on the left column correspond to subunits homologous to essential subunits in the mammalian Mediator complex. L in the A. fumigatus column indicates that uninfected controls succumbed at the same time rate as challenged flies, making it difficult to draw any conclusion with regards to susceptibility to this challenge. ?: the phenotype is not certain as mock-infected controls displayed some sensitivity to the infection procedure. The column next to the right-most column recapitulates the developmental phenotypes shown in Table 1: ++: no escapers; +: some pupal escapers that did not reach the adult stage; +/-: some adult escapers. The right-most column shows the results of monitoring the steady-state transcripts of the targeted gene; -: efficient RNAi with few transcripts detected; +/-: partial depletion of transcripts. Similar results were obtained by “classical” RTqPCR, which is, however, not as precise. /: not tested. Med subunit in bold: the RNAi line is unlikely to function efficiently.

## Discussion

**Figure 7:**
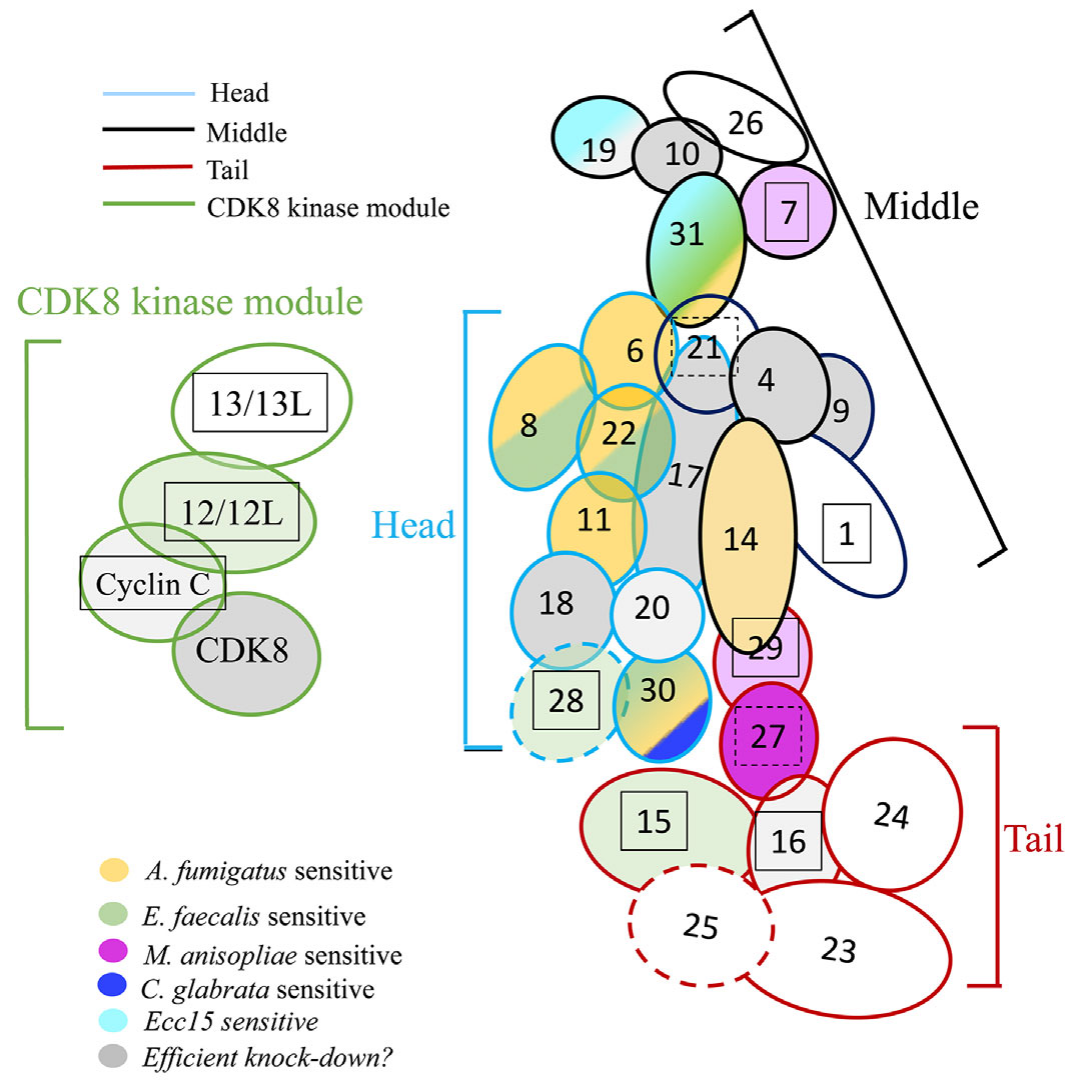
Roles of Mediator complex modules against infectious pathogens. The Mediator complex is composed of a central complex and of the CDK kinase module (CKM). The central complex has three modules: the head (light blue circumference), the middle (black circumference), and the tail (red circumference). Med14 constitutes a scaffolding subunit indicated by dark blue. The CKM (green circumference) consists of four subunits: *CDK8* (or its paralog *CDK19*), *Med12* (or its paralog *Med12L*), *Med13* (or its paralog *Med13L*), and Cyclin C. Subunits sensitive to *A. fumigatus* are shown in yellow; subunits sensitive to *E. faecalis* in green, light green when the phenotype is uncertain due to a lethality observed in the *A. fumigatus* experiments (Med12); subunits sensitive to *M. robertsii* natural infection are displayed in dark purple, light purple when the phenotype is uncertain; subunits sensitive to *M. robertsii* injection are shown in orange (only *Med17* and *Med31* RNAi flies have been tested); subunits sensitive to *C. glabrata* infection are pictured in dark blue whereas subunits sensitive to *Ecc15* infection are light blue. Lines for which the RNAi may not be effective are displayed in gray; a lighter gray indicates lines in which pupae developed upon continuous expression of the RNAi transgene during development, yet did not yield any adult flies (metamorphosis phenotype). Lines that yielded a lethal phenotype in the uninfected controls in the *A. fumigatus* experiments when gene expression was inhibited in the adult display a boxed subunit number, which is dashed when sensitivity was observed in the wounding (PBS-injected) but not the uninfected controls.

We have tested the roles of almost all the subunits of Mediator complex in host defense against infectious pathogens including a filamentous fungus (*A. fumigatus*), an entomopathogenic fungus in its filamentous form (*M. robertsii* natural infection; Wang *et al*., *in preparation*), or under the hyphal body form (*M. robertsii* conidia injection; Wang *et al*., *in preparation*), yeast (*C. glabrata*), a Gram-positive bacterium (*E. faecalis*), and a Gram-negative bacterium (*Ecc15*). All of the above-listed pathogens, except for *Ecc15*, are controlled, at least to some extent, by the Toll pathway. Our results showed that different Mediator subunits displayed distinct sensitivity to infections (**Figure 7**, **Figure S6**, **Table 4**) and reveal differential modes of actions of the Toll pathway.

A recent structural and genetic analysis of the Mediator complex in Mammals has revealed that 15 subunits are essential for cell viability in three human cell types (**Table 4**), mostly subunits of the core module (except for Med20 in the head module and Med1, Med9, Med19, Med26 in the middle module) and three tail subunits interfacing with the head and middle modules, namely Med27, Med28, and Med30 as well as the scaffolding subunit Med14. These essential subunits appear to be indispensable for the function of the Mediator complex in globally recruiting RNA Polymerase B to promoters to form the preinitiation complex (36). Here, we find that most subunits are required during development, except for Med4, Med9, Med10, Med18, Med19, Med20, CycC, and CDK8 for which some escaper pupae or adults were obtained (**Table 1**). It is likely that the *Med18* phenotype is due to a partial attenuation of its expression at the mRNA level as determined by regular and digital RTqPCR. We have not checked the efficiency of RNA interference for *Med16*, *Med19*, *CycC*, and *CDK8*, and thus cannot formally exclude a similar explanation for these subunits. In contrast, the RNAi approach seems to be efficient as regards *Med4*, *Med9*, *Med10*, and *Med20*. While Med9 and Med20 are also not indispensable in Mammals, Med4 and Med10 may in contrast be essential in Mammals but not in insects, although it is difficult to compare viability at the cellular *vs.* at the organismal level. Indeed, the depletion of Med subunits only at the adult stage led to the demise of eight uninfected RNAi mutant lines in the experiments in which susceptibility to *A. fumigatus* was tested. This occurred also when subunits not homologous to essential mammalian subunits were targeted, *i.e., Med1, Med12, Med13, Med15, Med16, Med28*, and *Med29*. It was unexpected that the uninfected controls for the same lines did not reveal any lethality when *E. faecalis* was tested later in another series of survival experiments (**Table 4**). This uninfected control was not performed for survival experiments with other tested pathogens; however, mock-infected controls were performed in these experiments and did not reveal any unusual viability issue when kept at 29°C, except where indicated in **Table 4** (*M. robertsii* natural infections). We suspect that conditions were slightly harsher in the *A. fumigatus* survival experiment series, resulting in a more efficient RNAi and depletion of the cognate subunit thus revealing their essential function, even in the context of the adult in which cells do not divide, somatic and germinal stem cells excepted. In contrast to classical mutations induced by chemical mutagenesis or CRISPR-Cas9, a fundamental difference with conditional RNAi is that the proteins are made in the cell prior to the induction of the RNAi. Even if the interference is 100% effective, the limiting factor to express the phenotype will be the relative stability of the protein already made, which may also vary from one target to the other, thus modulating the gamut of observable phenotypes. Indeed, it has already been noted in genome-wide genetic screens that the genes identified by RNA interference and those retrieved with random mutagenesis techniques differ extensively (37). Thus, this limitation has to be kept in mind when interpreting the results from this study.

One interesting observation relates to the CDK8 module: the Med12 and Med13 subunits appear to be essential in the adult, but not CycC and CDK8, even though the RNAi targeting each of the latter two subunits is effective, at least for *CycC* since no adults were retrieved when the RNAi transgene was expressed continuously throughout development. One possibility would be that the CycC and CDK8 proteins are more stable, although one should note that the survival experiments were monitored for over two weeks while the whole development at 29°C occurs in less than ten days. An alternative is that *Med12* and *Med13* have differing functions from the Cdk8/CycC kinase module of the Mediator complex, in keeping with a previous study (38).

We found that mutations affecting some of the Mediator complex subunits were not sensitive to any pathogen we have tested: Med4, Med9, Med10, Med20, Med26 of the core module, Med23, Med24, Med 25 of the tail module, and Med13 and CycC for the CDK8 kinase module (**Figure 7**, **Figure S6**, **Table 4**). Most of these subunits correspond to nonessential subunits in the mammalian complex, except for Med4 and Med10. Med20 is the only subunit of the head module not playing a role in the host defense against the pathogens we have tested and consistently is also the only subunit of the head not essential in the mammalian complex (36).

The only RNAi lines displaying a sensitivity to *A. fumigatus* infection affect the expression of genes encoding subunits of the head module with two exceptions, Med31 in the middle module and Med30 in the tail module (**Figure 7**, **Figure S6**, **Tables 2**, **4**). With respect to *E. faecalis* infection, the RNAi susceptible lines correspond to many subunits of the head module (Med8, Med17, Med22, Med28). We cannot formally exclude that Med6 and Med11 would also have tested positive had the conditions been as stringent as for the *A. fumigatus* infections. Beyond the head module, Med15, Med28, and Med30 in the tail module and Med31 in the middle module also displayed an *E. faecalis* infection sensitivity phenotype (**Figure 7**, **Figure S6**, **Tables 3**, **4**). As the *Med15* and *Med28* RNAi lines uninfected flies displayed a lethal phenotype in the *A. fumigatus* survival experiment series, we cannot exclude that these subunits may also be required for host defense against this fungus. It follows that it is an open possibility that host defense against these two pathogens involves the same set of Mediator subunits (**Figure 7**, **Table 4**).

*Med30* expression down-regulation was the only one that led to a sensitivity to *C. glabrata* infection and corresponds to a subunit of the tail module of Mediator also required for host defense against *A. fumigatus* and *E. faecalis* (**Figure 7**, **Figure S6**, **Tables 3**, **4**). This finding suggests that host defense against *C. glabrata* is strikingly distinct from that of the two microbes discussed so far. Even though DIF plays a central role in host defense against some fungal and Gram-positive bacterial infections, it is not required in the host defense against *C. glabrata* and its involvement in the defense against *A. fumigatus* has not been reported yet (6, 28, 39).

Only the *Med19* RNAi line displayed sensitivity to *Ecc15* infection (**Figure 7**, **Figure S6**, **Tables 3**, **4**). It is not clear at this stage whether this phenotype reflects an impaired function of the IMD pathway in the case of *Med19*. We expected to find a role for Med25 as it has been identified as being required for IMD pathway activation in an RNAi screen performed on cultured cells (40). This observation suggests that the regulation of the IMD pathway in cultured cells and *in vivo* may not be identical, at least as regards the role of the Mediator complex.

Med30 and Med31 display unique phenotypes, in that they are required for host defense against three types of infections, including *E. faecalis* and *A. fumigatus*. *Med30* RNAi flies are the only ones to be sensitive to *C. glabrata* whereas *Med31* flies are susceptible to injected *M. robertsii* conidia but not in a natural infection paradigm. Unexpectedly, distinct subunits appear to be involved in the response to this type of infection, in which the pathogen breaks through the cuticle, namely Med27 and possibly Med7 and Med29. This result is striking in that the Toll pathway and DIF are both required in the host defense against *M. robertsii* in either infection model (4, Wang et al., in preparation). Future work will tell whether the subunits required for host defense against *M. robertsii* in the natural infection model are required locally, for instance in the hypodermis or whether they mediate the action of DIF in the fat body. Of note, only two subunits, Med31 and Med17, have been tested in the *M. robertsii* conidia injection model (**Table 4**, **Figure S6**).

Med17 is the subunit shown to bind directly to DIF *in vitro* (25). DIF is required for the induction of multiple genes regulated by the Toll pathway (41), including *Drosomycin* and *BomS1/DIM1*. It was therefore surprising to observe that the induced *BomS1* expression was not impaired in *Med17* RNAi KD flies, although that of *Drosomycin* was affected. This situation is reminiscent of that documented for *Med1* during *Drosophila* development: it recruits GATA transcription factors only for a subset of genes regulated by these factors (24). Thus, it is likely that the context plays an essential role, not only with respect to the regulation of specific subsets of genes through a given set of Mediator subunits, but also depending on the pathogen and infection route. Indeed, there is only a limited overlap of genes with an altered expression in the natural infection or conidia injection models of *M. robertsii* injection. It should also be kept in mind that host defense is not limited to resistance, for instance through AMPs, but involves the dimension of resilience/tolerance whereby the host withstands or repairs damages exerted by pathogen virulence factors or its own immune response (42–44). As exemplified by the finding that the *Caenorhabditis elegans* Med15 is required for host defense against *Pseudomonas aeruginosa* and detoxification of some of the toxins it secretes such as phenazines (45), it will be therefore interesting to determine whether some of the Med subunits identified in this study for their involvement in host defense actually mediate resistance, resilience, or both.

## Data Availability Statement

The datasets generated for this study are available on request to the corresponding author.

## Author Contributions

CH performed most experiments described in this work and participated in the genetic screen that led to this work. RX developed the *A. fumigatus* infection model and started the genetic screen that led to the identification of Med subunit genes; he also characterized the Med17 RNAi phenotype and performed the digital PCR experiments. SL and ZL provided guidance and supervision for the experiments. DC and DF played a key role in designing the genetic screen, and DC organized the actual implementation of the genetic screen and coordinated the logistics. DF and CH designed the experiments, analyzed the data, and wrote the manuscript. All authors contributed to the article and approved the submitted version.

## Funding

This work was funded through the Incubation Project for Innovation Team of the Guangzhou Medical University (# B1850004105) and the 111 Project (# D18010) as well as support from the China 1,000 Talent program to DF.

## Conflict of Interest

The authors declare that the research was conducted in the absence of any commercial or financial relationships that could be construed as a potential conflict of interest.
