# Supplementary material for: Differential Requirements for Mediator Complex Subunits in Drosophila melanogaster Host Defense Against Fungal and Bacterial Pathogens
Source: Front Immunol. 2021 Mar 5;11:478958. doi: 10.3389/fimmu.2020.478958 (PMC7977287; doi:10.3389/fimmu.2020.478958)
Supplement: Supplementary file 1 [file DataSheet_1.pdf]

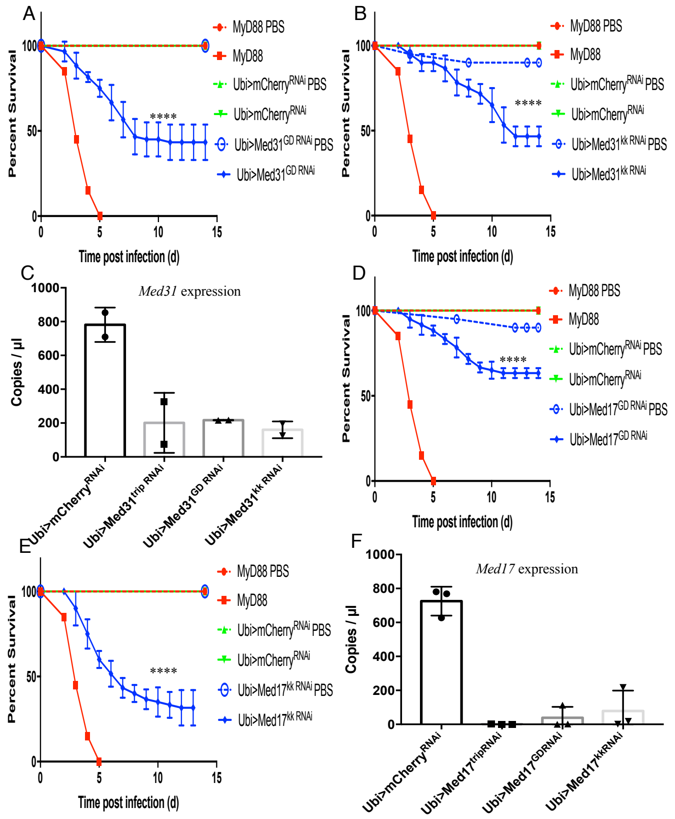

Supplementary Figure 1: Further independent RNAi lines targeting *Med31* or *Med17* are susceptible to *A. fumigatus* infection

Survival of *Med31* GD and KK RNAi flies (A-B) and of *Med17* GD and KK RNAi flies (D-E) after *A. fumigatus* infection. *MyD88* represents the positive control line (red), Ubi>*mCherry* RNAi line represents the wild type control line (green), and the Ubi> RNAi line of Mediator complex subunits is shown in blue. Each infected line has a PBS-injected control (dashed lines). Both GD and KK lines of *Med31* and *Med17* succumbed faster to infection than the *mCherry* RNAi controls. Infected Mediator complex subunits RNAi flies *vs.* infected *mCherry* RNAi flies: log-rank test, ****, P<0.0001. These experiments were performed three times. C, F: the RNAi efficiency of three RNAi lines targeting *Med31*(C) and *Med17*(F), which was measured using digital RTqPCR. Mean ±SEM are shown.

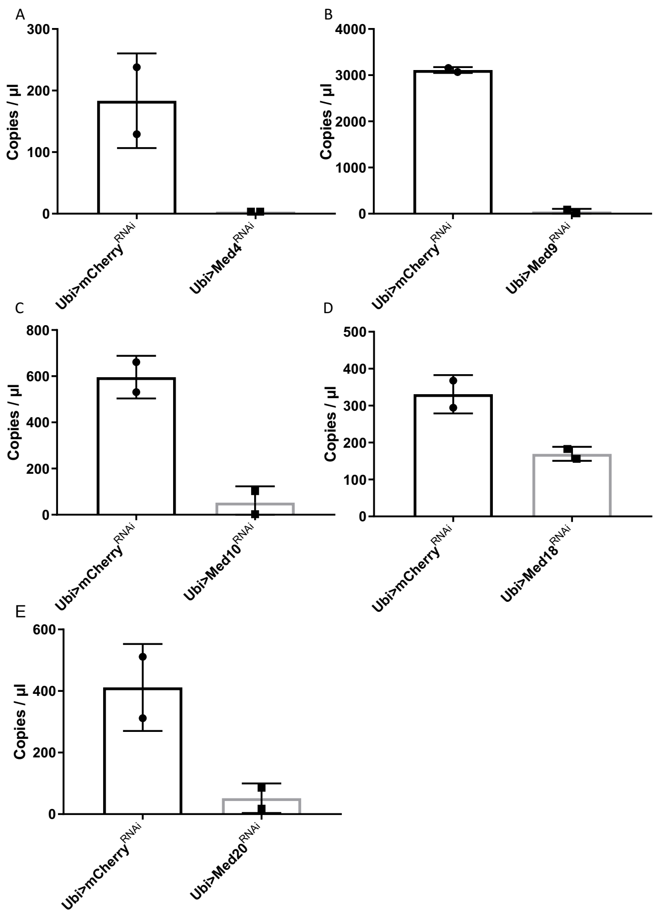

Supplementary Figure 2: Validation of the RNAi lines displaying a mild developmental effect when Ubi >Med RNAi were raised at 29°C (Table 1)
The RNAi efficiency of *Med4* (A), *Med9* (B), *Med10* (C), and *Med20* (E) were validated by digital RT-qPCR, except for *Med18* (D). Each dot represents one sample containing five flies. Mean ±SEM are shown.

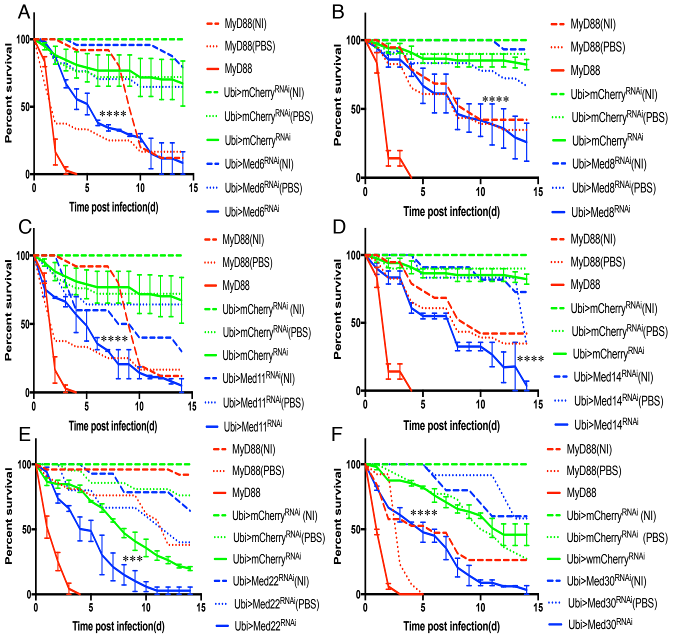

Supplementary Figure 3: *A. fumigatus* infection susceptibility phenotypes for other Mediator subunit-encoding genes.

Survival of RNAi flies of *Med6* (A), *Med8* (B), *Med11* (C), *Med14* (D), *Med22* (E) and *Med30* (F) after *A. fumigatus* infection. *MyD88* represents the positive control line (red), Ubi>*mCherry* RNAi line represents the wild type control line (green) and Ubi> RNAi line of Mediator complex subunits (blue). Each infected line has a non-infected (NI: dashed line)) and PBS injected control (dotted lines). All RNAi flies succumbed faster to infection than the *mCherry* RNAi controls. Infected Mediator complex subunits RNAi flies *vs.* infected *mCherry* RNAi flies: log-rank test, ***, P<0.001, ****, P<0.0001. These experiments were performed at least three times. Mean ±SEM are shown.

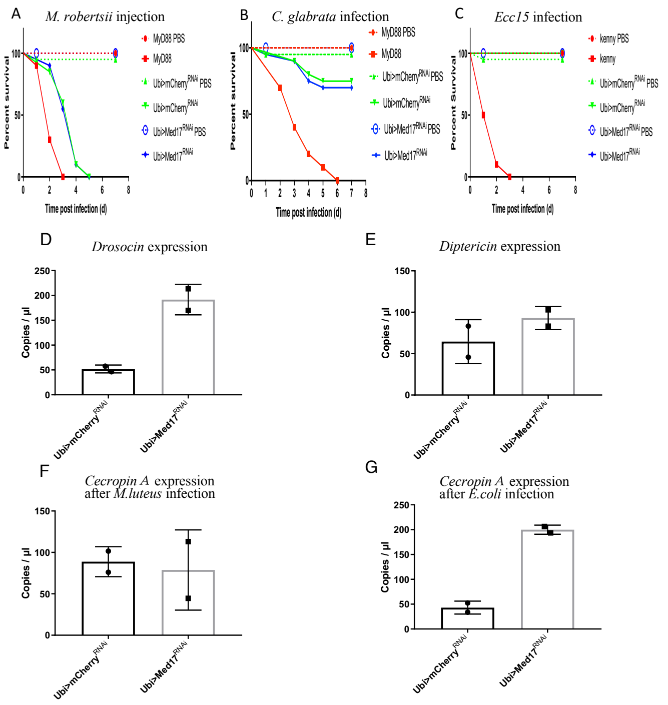

Supplementary figure 4: Lack of susceptibility of *Med17* RNAi flies to three microbial challenges

Survival of Ubi > *Med17* RNAi flies to other pathogen challenges and expression of AMP genes in Ubi > *Med17* RNAi flies after challenge with *M. luteus* or *E. coli*. There was no significant difference between Ubi > *Med17* RNAi flies and Ubi > *mCherry* RNAi flies after *M. robertsii* injection (A), *C. glabrata* infection (B) and *Ecc15* infection (C). Digital RTqPCR data showed that *Drosocin* (D), *Diptericin* (E) and *CecropinA* (G) expression levels have a trend toward higher expression in Ubi > *Med17* RNAi flies than in Ubi > *mCherry* RNAi flies after *E. coli* infection; in contrast, *CecropinA* expression levels did not vary much after *M. luteus* infection (F).

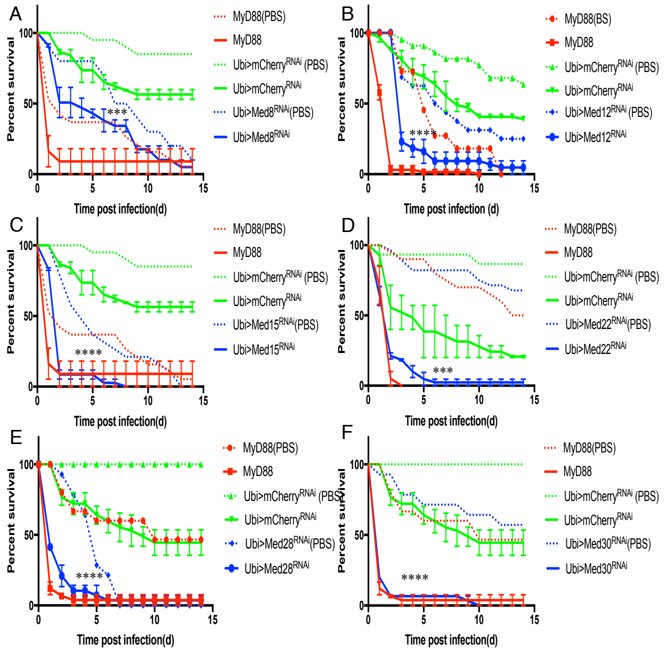

Supplementary Figure 5: Other Mediator complex subunits susceptible to *E. faecalis* infection

Survival of RNAi flies of *Med8* (A), *Med12* (B), *Med15* (C), *Med22* (D), *Med28* (E) and *Med30* (F) after *E. faecalis* infection. *MyD88* represents the positive control line (red), Ubi>*mCherry* line was the wild type control line (green), and Ubi> RNAi line of Mediator complex subunits is displayed in blue. Each infected line has a PBS injected control (dotted lines). RNAi flies of *Med8*, *Med12, Med15, Med22*, *Med28* and *Med30* succumbed faster to infection than the Ubi>*mCherry* controls. Of note, *Med15* and *Med28* RNAi flies are susceptible to PBS-injection. The demise of these lines after *E. feacalis* injection correlates however with a higher bacterial burden (not shown). Infected Mediator complex subunits RNAi flies *vs.* infected Ubi>*mCherry* flies: log-rank test, ***, P<0.001; ****, P<0.0001. All the experiments were performed at least twice, except for *Med12*, which was done only once. Mean ±SEM are shown.

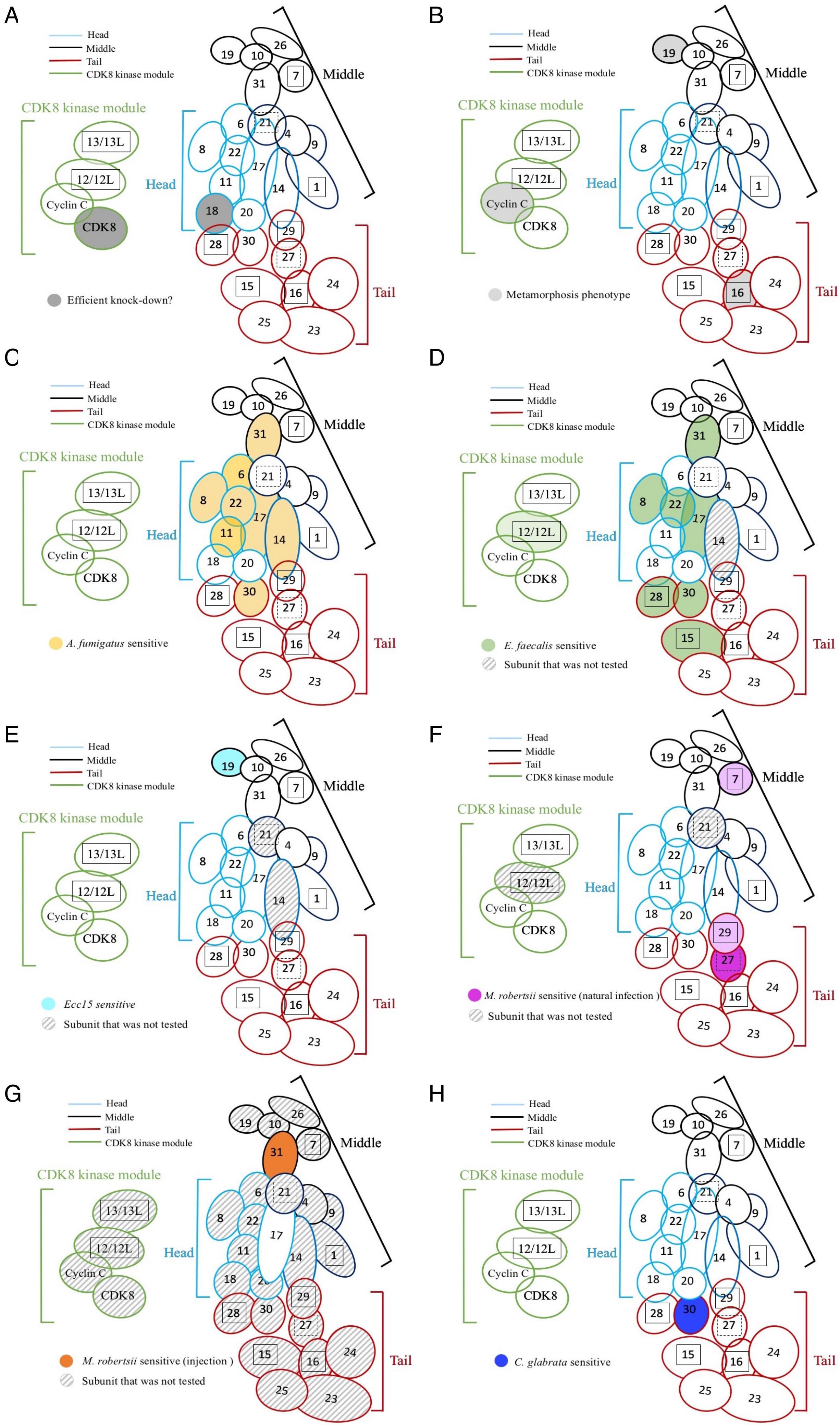

Supplementary figure 6: Susceptibility of Mediator complex subunits against tested pathogens.

Supplementary figure 6 *(continued)*. Susceptibility of Mediator complex subunits against tested pathogens.

A: Lines for which the RNAi may not be effective are displayed in gray; B: a lighter gray indicates lines in which escaper pupae developed upon continuous expression of the RNAi transgene during development, yet did not yield any adult flies (metamorphosis phenotype); C: Subunits sensitive to *A. fumigatus* are shown in yellow; D: subunits sensitive to *E. faecalis* are shown in green, light green when the phenotype is uncertain (Med12); E: subunits sensitive to *Ecc15* infection are displayed in light blue; F: subunits sensitive to *M. robertsii* natural infection are shown in dark purple, light purple when the phenotype is uncertain; G: subunits sensitive to *M. robertsii* injection are displayed in orange; H: the subunit sensitive to *C. glabrata* infection is highlighted in dark blue. Lines that yielded a lethal phenotype in the uninfected controls in the *A. fumigatus* experiments when gene expression was inhibited in the adult are displayed with a boxed subunit number, which is dashed when the sensitivity was observed in the wounding (PBS-injected) but not the uninfected controls. Lines which were not tested are indicated by gray hatching.

	Mediator complex subunits	CG#	stock#
1	Med 1	CG7162	v13054
2	Med 4	CG8609	THU3678
3	Med 6	CG9473	THU3629
4	Med 7	CG31390	THU3664
5	Med 8	CG13867	THU1508
6	Med 9	CG42517	BL33678
7	Med 10	CG5057	THU1384
8	Med 11	CG6884	THU3631
9	Med 12	CG8491	THU5188
10	Med 13	CG9936	THU1534
11	Med 14	CG12031	THU3617
12	Med 15	CG4184	THU0962
13	Med 16	CG5465	THU1361
14	Med 17	CG7957	BL34664<br>v44027<br>v105264
15	Med 18	CG14802	BL42634
16	Med 19	CG5546	THU1019
17	Med 20	CG18780	THU3619
18	Med 21	CG17397	THU1459
19	Med 22	CG3034	THU3616
20	Med 23	CG3695	TH02351.N
21	Med 24	CG7999	THU3632
22	Med 25	CG12254	THU4907
23	Med 26	CG1793	THU4872
24	Med 27	CG1245	THU3618
25	Med 28	CG5121	THU0904
26	Med 29	CG13201	TH03883.N
27	Med 30	CG17183	THU1802
28	Med 31	CG1057	THU5186<br>v27285<br>v101488
29	CDK8	CG10572	v31264
30	Cyc C	CG7281	v27937

Supplementary Table 1. List of RNAi fly stocks.

Primers	Sequences
rp49-F	GACGCTTCAAGGGACAGTATCTG
rp49-R	AAACGCGGTTCTGCATGAG
Drs-F	CGTGAGAACCTTTTCCAATATGAT<br>G
Drs-R	TCCCAGGACCACCAGCAT
DIM1-F	CAATGCTGTTCCACTGTCGC
DIM1-R	CGTGGACATTGCACACCCTG
Dpt-F	GCTGCGCAATCGCTTCTACT
Dpt-R	TGGTGGAGTGGGCTTCATG
CecA-F	ACGCGTTGGTCAGCACACT
CecA-R	ACATTGGCGGCTTGTTGAG
Dros-F	TGAAGTTCACCATCGTTTTCCTG
Dros-R	CACCCATGGCAAAAACGC
Med 4-F	AGGAGGCGATGGACCAACT
Med 4-R	GCATCCTTTAGCGACTTTTGC
Med 9-F	GGACATTGAGATTCTGCCGATAA
Med 9-R	GCTTAACGGCGTTCTCCAGAG
Med 10-F	TGCACCGCTAGAGAACCTTG
Med 10-R	CTTGTAGGCCGGTCACCAAG
Med 17-F	ACGACTTGCGAGAACCAGATA
Med 17-R	TTTCGCCAGACTTTCCGACAA
Med 18-F	CATGCGATGAACAACCGCTT
Med 18-R	GTGCATCAAGTGTTCCACGG
Med 20-F	TCATGCTCAAGATGACCAACAC
Med 20-R	ACAAAGTCACCATACTCGAAACG
Med 31-F	CACAGCGTGGATTTTTCAAGG
Med 31-R	GTATTTCGCATAGTCCGGCTC

Supplementary Table 2. Primers used forRT-qPCR and digital PCR
